# The iNKT Cell–Macrophage Axis in Homeostasis and Disease

**DOI:** 10.3390/ijms23031640

**Published:** 2022-01-31

**Authors:** Mariana S. Cruz, José Pedro Loureiro, Maria J. Oliveira, Maria Fatima Macedo

**Affiliations:** 1 Cell Activation and Gene Expression Group, Instituto de Biologia Molecular e Celular (IBMC), Instituto de Investigação e Inovação em Saúde (i3S), Universidade do Porto, Rua Alfredo Allen 208, 4200-135 Porto, Portugal; marianascruz@ua.pt (M.S.C.); loureiro9jpedro@gmail.com (J.P.L.); 2 Department of Medical Sciences, University of Aveiro (UA), 3810-193 Aveiro, Portugal; 3 Experimental Immunology Group, Department of Biomedicine (DBM), University of Basel and University Hospital Basel, Hebelstrasse 20, 4031 Basel, Switzerland; 4 Tumour and Microenvironment Interactions Group, Instituto Nacional de Engenharia Biomédica (INEB), Instituto de Investigação e Inovação em Saúde (i3S), Universidade do Porto, Rua Alfredo Allen 208, 4200-135 Porto, Portugal; mariajo@ineb.up.pt; 5 Department of Molecular Biology, ICBAS-Institute of Biomedical Sciences Abel Salazar, Universidade do Porto, Rua Jorge Viterbo Ferreira 228, 4050-313 Porto, Portugal

**Keywords:** NKT cells, CD1d, macrophages, immune crosstalk, cell activation

## Abstract

Invariant natural killer T (iNKT) cells are CD1d-restricted, lipid-reactive T cells that exhibit preponderant immunomodulatory properties. The ultimate protective or deleterious functions displayed by iNKT cells in tissues are known to be partially shaped by the interactions they establish with other immune cells. In particular, the iNKT cell–macrophage crosstalk has gained growing interest over the past two decades. Accumulating evidence has highlighted that this immune axis plays central roles not only in maintaining homeostasis but also during the development of several pathologies. Hence, this review summarizes the reported features of the iNKT cell–macrophage axis in health and disease. We discuss the pathophysiological significance of this interplay and provide an overview of how both cells communicate with each other to regulate disease onset and progression in the context of infection, obesity, sterile inflammation, cancer and autoimmunity.

## 1. Introduction

Natural Killer T (NKT) cells constitute a distinct population of circulating and tissue-resident effector T cells that are CD1d-restricted and whose T-cell receptor (TCR) recognizes lipid antigens. CD1d is a non-polymorphic major histocompatibility complex (MHC) class I-like protein expressed by antigen-presenting cells (APCs) and is required for lipid antigen presentation to NKT cells [1,2,3]. NKT cells can be subdivided into two distinct populations according to their TCR repertoire: (i) type I, or invariant NKT (iNKT) cells, which express a semi-invariant TCR; and (ii) type II, characterized by TCR polyclonality [1]. In mice, iNKT TCR uses the Vα14Jα18 chain paired with a limited number of Vβ chains, which are typically Vβ2, Vβ7, or Vβ8, whereas in humans, iNKT TCR is predominantly Vα24Jα18/Vβ11 [1,4,5,6]. Unlike type II NKT cells, iNKT cells are also characterized by their reactivity to the lipid antigen α-Galactosylceramide (α-GalCer) [2], hence frequently detected through the usage of CD1d dimers or tetramers loaded with α-GalCer or analogues [7,8]. In addition, human iNKT cells can also be detected based on their double positivity for anti-Vα24 and anti-Vβ11 antibodies [7,8,9]. However, this approach exhibits some limitations, given that there are reports of non-invariant, non-CD1d-restricted Vα24^+^Vβ11^+^ cells [9]. Hence, to surpass this lack of specificity, another approach has been developed to identify human iNKT cells, consisting of the 6B11 antibody, which is specific for the CDR3 region on the semi-invariant TCR [8,9].

iNKT cells originate in the thymus, arising from the same lymphoid precursors as MHC-restricted T cells, and are mainly non-circulating, tissue-resident cells [1,10,11]. However, unlike conventional T cells, iNKT cells migrating from the thymus already exhibit a memory/effector phenotype [12]. Murine iNKT cells are classified into three distinct functional subsets (NKT1, NKT2 and NKT17), resembling the well-described Th1, Th2 and Th17 subpopulations of CD4^+^ T cells [10].

iNKT cells recognize both endogenous and exogenous lipids and are strongly activated in a TCR-dependent manner upon recognition of lipid-loaded CD1d complexes [10,13]. In addition, iNKT cell activation can also occur in a TCR-independent manner in response to APC-derived IL-12 and IL-18 [14,15,16,17]. Upon activation, iNKT cells display innate-like properties and rapidly secrete great amounts of Th1- and Th2-like cytokines, including IFN-γ, IL-4 and GM-CSF, which may, in turn, modulate APC activation [12,13,18]. Along with their diverse cytokine profile, iNKT cells also exhibit potent cytolytic activity supported by the release of cytotoxic granules containing perforin and granzyme or by activation of death receptor pathways involving Fas-FasL and TRAIL-DR5 interactions [19,20,21]. Furthermore, iNKT cells trigger transactivation of other immune cells, including natural killer (NK) cells, macrophages, dendritic cells (DCs), and B and T lymphocytes [15,22,23,24,25]. Hence, the ultimate protective or pathological functions displayed by iNKT cells in tissues are shaped by the pro- or anti-inflammatory nature of their secreted cytokines, their cytotoxic functions and the interactions they establish with other immune cells [12,18].

More specifically, iNKT cell–macrophage crosstalk has gained growing interest in the past decades. Macrophages are remarkably plastic and heterogeneous cells whose activation state, phenotypic signature and inflammatory profile change in response to distinct signaling cues in the local microenvironment [26,27,28]. Due to this, macrophages fall within a continuum spectrum of activated phenotypes [29]. Nevertheless, two distinct macrophage populations are frequently considered: (i) M1-like macrophages, characterized by strong secretion of pro-inflammatory cytokines (such as IL-12, IL-6, TNF-α and IL-1β), Th1-attracting chemokines and reactive oxygen and nitrogen species [26,27,28,30,31]; and (ii) M2-like macrophages, exhibiting an anti-inflammatory profile marked by production of the immunosuppressive cytokines IL-10 and TGF-β, the chemokines CCL17, CCL18 and CCL22, and extracellular matrix-related proteins [26,28,32,33]. Murine macrophages are generally identified according to their expression of distinct cell surface markers, including CD11b, CD68, F4/80 and Ly-6C, whereas human macrophages exhibit expression of CD14, CD11b, CD16 and CD68, among others [34,35]. Macrophages are critically involved in inflammation, pathogen clearance, tissue repair and immunomodulation and seem to play bimodal roles in health and disease [12,28,36]. Importantly, it is becoming increasingly evident that communication between iNKT cells and macrophages may play deleterious or protective roles in several diseases. Hence, this review will cover the pathophysiological significance of the iNKT cell–macrophage axis, particularly in the context of infection, obesity, sterile inflammation, cancer and autoimmunity.

## 2. The iNKT Cell–Macrophage Axis

The crosstalk between iNKT cells and CD1d-expressing myeloid cells is supported not only by the engagement of the semi-invariant TCR with lipid-loaded CD1d molecules expressed at the APC surface but also by the co-stimulatory axes of CD40-CD40L and CD80/CD86-CD28 and by pro- and anti-inflammatory cytokines secreted by both cells [20,37,38].

Importantly, the nature of the iNKT cell–macrophage intercellular communication is bidirectional. Firstly, as competent APCs, macrophages can present lipid antigens loaded on CD1d molecules to activate iNKT cells, ultimately modulating the immune responses displayed by iNKT cells in tissues. On the other hand, activated iNKT cells are simultaneously able to modulate macrophage activation and polarization, altering their phenotypic and functional properties [39]. The latter effect is mostly dependent on the panel of cytokines secreted by activated iNKT cells [39].

### 2.1. The iNKT Cell–Macrophage Axis in Homeostasis

Key features of lipid antigen presentation by macrophages to iNKT cells in steady state have been reported in both primary (thymus) and secondary lymphoid (lymph nodes, spleen and mucosal tissues), as well as in non-lymphoid tissues, including the liver and the adipose tissue.

Regarding the thymus, a recent report described that TCR engagement by CD1d-expressing macrophages assures steady-state IL-4 production by NKT2 cells in the thymic medulla [35]. By selectively abrogating CD1d expression on thymic CD11c^+^ cells or depleting thymic F4/80^+^ Mertk^+^ macrophages through a diphtheria toxin-based approach, Wang et al. observed significant decreases in NKT2 abundance and IL-4 production by these cells in thymus medulla. These experiments provided evidence for the pivotal roles of macrophages in the activation and acquisition of effector functions by thymic iNKT cells [40].

Accumulating evidence has reinforced that macrophages also serve as crucial APCs to modulate iNKT cell activation in secondary lymphoid tissues. In mice, adoptively transferred iNKT cells were shown to preferentially populate the paracortical region of lymph nodes [41]. There, subcapsular sinus (SCS) CD169^+^ macrophages were reported to retain, internalize and present particular lipid antigens in a CD1d-restricted manner to iNKT cells. Hence, lymph-node-resident macrophages seem to be critical participants in iNKT cell early activation, cytokine secretion and expansion in the lymph nodes [41]. Accordingly, in vivo targeted delivery of liposomes containing α-GalCer to CD169^+^ macrophages via CD169-mediated endocytosis prompted strong IFN-γ and IL-4 production by splenic and hepatic iNKT cells [42]. In fact, depletion of CD169^+^ macrophages prevented α-GalCer-driven iNKT cell activation in both the spleen [42] and draining lymph nodes [41,43].

In the spleen, iNKT cells (endogenous or adoptively transferred) locate to both splenic-red-pulp (RP) and marginal-zone (MZ) compartments [44]. However, iNKT cells have been shown to relocate preferentially into the antigen-rich MZ upon intravenous administration of α-GalCer or GalA-GSL (an iNKT cell-glycosphingolipid antigen expressed by Sphingomonas bacteria), either in soluble [45] or particulate form [44]. This relocation to the MZ did not occur after IL-12 or IL-18 administration [45]. Whereas lipid immunization activated splenic iNKT cells to produce IFN-γ and IL-4, this effect was diminished following depletion of splenic CD11c^+^ cells, including both DCs and macrophages [44]. Splenic DCs and MZ SIGN-R1^+^ macrophages express CD1d and are capable of presenting particulate lipids in vitro to induce iNKT cell activation [44].

On another note, expression of proteins associated with lipid homeostasis by splenic macrophages is also suggested as a factor contributing to macrophage-mediated activation of iNKT cells in the spleen [46]. Splenic F4/80^+^ macrophages exhibit the highest expression levels of low-density lipoprotein-receptor-related protein (LRP), which is involved in lipoprotein metabolism, in comparison with DCs, B cells and T cells [46]. Although macrophage-specific LRP deficiency did not affect iNKT cell activation in vitro, it impaired in vivo α-GalCer-stimulated splenic iNKT cells to secrete IL-4 but not IFN-γ [46]. Altogether, these data point to macrophages playing fundamental roles in iNKT cell activation in secondary lymphoid tissues.

iNKT cells are known to be crucial regulators of mucosal immunity [47,48], and recent research has emphasized the contribution of intestinal APCs in the immunomodulatory roles of iNKT cells. Intestinal CD1d-expressing CD11c^+^ myeloid cells, comprising both DCs and macrophages, present lipids to activate gut-associated iNKT cells, differentially controlling the abundance of each iNKT cell subset in the intestine [49]. Moreover, intestinal bacteria composition and compartmentalization, as well as IgA repertoire, differed between CD1d^−/−^ mice and WT or CD1d^+/−^ littermate controls, thus indicating that iNKT cells and CD1d expression control intestinal microbiota and homeostasis [49]. Supporting the relevance of the interactions between iNKT cells and CD1d-expressing myeloid cells in the intestine, Gensollen et al. recently reported that murine embryonic-, and non-bone-marrow-derived macrophages regulate the local establishment of iNKT cells in the colon during early periods of life, associated with susceptibility or resistance to iNKT cell-associated mucosal disorders in later life [50]. In transgenic mouse models in which macrophages express the diphtheria toxin receptor (DTR), depletion of splenic and colonic macrophages through administration of the diphtheria toxin resulted in reduced numbers and proliferative capacity of splenic and colon-resident iNKT cells. Moreover, macrophage-mediated regulation of colonic but not of splenic iNKT cell abundance was temporally restricted to the first 11 days post-birth [50].

Distinct roles of this interplay have also been described under homeostatic conditions in non-lymphoid tissues, including the liver and the adipose tissue. Both iNKT cells and macrophages can be found in the liver. Although in mice, iNKT cells are particularly enriched in the liver [1,10], their abundance in the human liver is not elevated [10]. On the other hand, liver-resident Kupffer cells (KCs) are specialized macrophages that originate from the yolk sac during embryonic development and represent 80–90% of tissue-resident macrophages in the body [51,52]. Considering the preferential distribution of mouse iNKT cells and KCs in the liver, one could hypothesize that the macrophage–iNKT cell axis may also be particularly relevant in this organ. Indeed, CD1d-expressing KCs have been identified as key APCs in the activation of iNKT cells in the liver [53]. KC depletion through clodronate liposome injection impaired α-GalCer-stimulated IL-4 and IFN-γ production by hepatic but not by splenic iNKT cells [53], once again underpinning that liver-tissue-resident macrophages present lipid antigens to activate iNKT cells.

Finally, adipose-tissue-resident iNKT cells were demonstrated to modulate macrophage phenotype. Adipose tissue iNKT cells and macrophages co-localized following α-GalCer immunization [54]. A-GalCer stimulation resulted in increased numbers of adipose-resident CD206^+^ and CD301^+^ M2 macrophages via iNKT-derived IL-10 [54]. Thus, IL-10-producing iNKT cells promote M2 polarization in steady-state adipose tissue. Supporting these iNKT-mediated effects on macrophage polarization, other researchers have described that cytokines secreted by iNKT cells differently regulate the inflammatory properties of macrophages. When co-culturing murine iNKT cells and peritoneal macrophages, GM-CSF secreted by iNKT cells upon activation induced macrophages to produce IL-1β, whereas iNKT-derived IL-4 had the opposite effect of decreasing IL-1β production by macrophages [55].

Collectively, these data unravel the importance of the communication between iNKT cells and macrophages in maintaining tissue immunological homeostasis.

### 2.2. The iNKT Cell–Macrophage Axis in Infection

**Figure 1:**
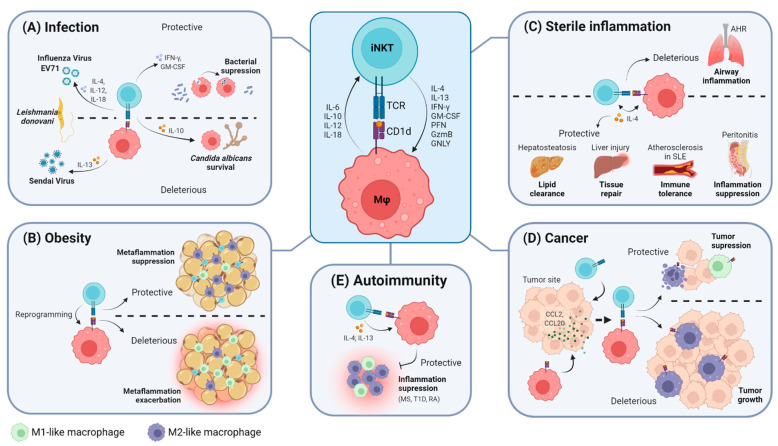
iNKT cells and macrophages interact under different pathological contexts. This crosstalk primarily involves the recognition of antigen-loaded CD1d molecules by the iNKT cell TCR, followed by the exchange of cytokines and the interplay of other effector molecules that are specific to each paradigm. (**A**) The outcome of interactions between iNKT cells and macrophages in infectious environments. (**B**) The outcome of interactions between iNKT cells and macrophages in obesity. (**C**) iNKT cells and macrophages interact differently under distinct sterile inflammatory conditions. (**D**) The iNKT cell–macrophage axis in the context of cancer. (**E**) The outcome of the interplay between iNKT cells and macrophages in autoimmunity. Created with BioRender.com in 22 January, 2022. PFN: perforin; GzmB: granzyme B; GNLY: granulysin; MS: Multiple sclerosis; T1D: type I diabetes; RA: rheumatoid arthritis.

In the context of infection, there is no consensus regarding the outcome of iNKT cell–macrophage interactions since it seems to be highly dependent on the aggressor. Data suggest that this crosstalk relies mostly on antigen presentation by CD1d and, in some cases, on innate-like mechanisms that will be further discussed and summarized in Table 1 and Figure 1A.

iNKT cells were proposed to alter macrophage phagocytic capacity [56] and phenotypic profiles [57] of peritoneal macrophages in murine models of sepsis. Heffernan et al. described that iNKT cells migrated from the liver into the peritoneum, tuning phagocytic functions of macrophages. It is worth mentioning that this migration was reported to be affected by programmed cell death-1 receptor (PD-1) expression [56]. PD-1 was later confirmed as a checkpoint for iNKT cells that influences phenotypic changes in peritoneal macrophages in another septic mouse model. After sepsis, PD-1-driven iNKT cells induce low expression of Ly6C, a marker of macrophage activation and transmigration ability, whereas the absence of these T cells led to high Ly6C expression [57]. In another mouse model for septic shock, iNKT cells were shown to be reduced in the liver, although they were more activated. Additionally, sepsis induced higher expression of IL-6 and IL-10 by liver macrophages, with in vivo blocking of CD1d leading to reduction of IL-6 and augmentation of IL-10 systemic levels, further improving survival rates [58].

The IL-10/iNKT axis seems to have an opposite effect in other infections. Infection by *Candida albicans* was shown to be more severe in the presence of activated iNKT cells [59]. Haraguchi et al. demonstrated that mice lacking iNKT cells turned out to be more resistant to the development of lethal candidiasis. These mice exhibited increased numbers of infiltrating phagocytic cells that secreted high concentrations of IL-1β and IL-18, contrasting with the high levels of IL-10 reported in WT mice [59]. Indeed, in vitro co-cultures validated that the production of IL-10 from infected macrophages was increased in the presence of iNKT cells, whereas the production of IL-1β and IL-18 was diminished. This phenomenon was ultimately confirmed, as susceptibility to *C. albicans* in Ja18 KO mice was restored following adoptive transfer of iNKT cells or IL-10 administration, indicating that iNKT cells modulate IL-10 production by macrophages [59]. Furthermore, in cases of *C. albicans* infection post-sepsis, in both mice and patients, iNKT cells were suggested to impair macrophage phagocytic capacity due to increased production of IFN-γ [60]. Transcriptomic analysis and in vivo experiments clarified that iNKT cells induced activation of mTORC1 signaling in NK cells, increasing IFN-γ production. At last, macrophage phagocytosis was suppressed, worsening both clearance of secondary infection and survival [60].

The outcome upon *Leishmania donovani* infection depends highly on the origin of the macrophages. Beattie et al. described enhanced iNKT cell activation in the presence of infected KCs [61], whereas Karmakar et al. reported an impairment of this activation in infected splenic macrophages [62,63]. In both reports, interesting mechanisms were enlightened by the authors. In mice, infection of KCs by *L. donovani* was observed to enhance hepatic iNKT cell activation by a mechanism that involved the interaction of signal-regulatory protein α (SIPRα) and the “don’t eat me” receptor, CD47 [61]. Using a CD47 KO mouse model, Beattie et al. showed that hepatic iNKT cells had a defective response to *L. donovani* infection, producing lower levels of IFN-γ [61]. Oppositely, splenic macrophages infected with *L. donovani* failed to activate iNKT cells [62]. This impairment could be corrected by treating cells with glycosphingophospholipid (GSPL), a stimulatory *L. donovani* antigen, in a process that required presentation by CD1d [62]. Later, it was also demonstrated that GSPL could simultaneously bind TLR4 on the APC, inducing IFN-γ production [63]. These data show that CD47 stands as an important player in hepatic iNKT cell activation [61], whereas TLR4 is crucial for splenic iNKT activation [63].

Another mechanism of KC–iNKT cell interaction was studied in a *Borrelia burgdorferi* infected mouse model. In this model, KCs induced iNKT cell clustering mediated by the chemokine receptor CXCR3 [64]. This crosstalk depended on CD1d and culminated in iNKT cell activation. The absence of iNKT cells caused a more efficient dissemination of *B. burgdorferi*, which escaped immunosurveillance and survived in the liver parenchyma. These results highlighted the importance of both cells in reducing diffusion of *B. burgdorferi* [64]. Moreover, in mice infected with *Listeria monocytogenes*, iNKT cells and macrophages seemed to join forces and ameliorate listeriosis [65]. Herein, activation of iNKT cells via α-GalCer improved the phagocytic and bactericidal activities of peritoneal macrophages via IFN-γ and nitric oxide (NO) production. This bactericidal function could be abrogated or partially abolished by blocking IFN-γ or NO, respectively [65].

In the lungs, the macrophage–iNKT cell axis has been implicated in several airway infections caused by *Mycobacterium tuberculosis* (Mtb) [66,67,68], *Pseudomonas aeruginosa* [69], *Streptococcus pneumoniae* [70] and influenza virus [71,72]. Although distinct in vitro studies using mouse and human cells showed that iNKT cells were activated by Mtb-infected macrophages via CD1d, IL-12 and IL-18 [66,67,68], iNKT-mediated suppression of bacterial growth was shown to be CD1d-dependent but did not require IL-12, IL-18 or IFN-γ [68]. Instead, iNKT cells were reported to express granulysin [66] and secrete GM-CSF in a CD1d-dependent manner [68]. When tested *in vivo*, both transfer of NKT cells into infected mice [67] and GM-CSF alone [68] were sufficient to control Mtb growth. Similarly, in a mouse model of pneumonia due to *P. aeruginosa* infection, α-GalCer-driven activation of iNKT cells enhanced phagocytosis by alveolar macrophages and consequent pathogen clearance [69]. In addition, an in vivo study of *S. pneumoniae* infection showed that Mrp1 deficiencies in macrophages reduced CD1d clustering at the cell surface, inducing lower activation of iNKT cells and resulting in increased mortality associated with the infection [70].

Activated iNKT cells were also shown to control influenza A infection through mechanisms involving recruitment of neutrophils, macrophages, monocytes and B cells [71,72,73]. Ho et al. demonstrated in mice that iNKT cell activation induced by α-GalCer enhanced early innate immune response, suggesting a possible migration of hepatic iNKT cells to the lungs upon α-GalCer administration [71]. In the same line, NKT cell activation was reported to result in an early wave of IL-4 secretion upon their relocation near lymph-node-resident macrophages and consequent priming. Induction of IL-4 required both CD1d and IL-18 [73]. Overall, iNKT cells were associated with a better survival rate in influenza-A-infected mice and efficiently killed human monocytes infected with influenza A [72]. Furthermore, development of Sendai virus (SeV)-induced chronic airway inflammation seemed to be potentiated by macrophage-derived IL-13 previously prompted by iNKT cells in mice [74]. Interestingly, in the lung tissue of patients with chronic airway inflammation, IL-13^+^ cells were identified as macrophages based on morphology and CD68 expression. CD68^+^IL-13^+^ cells were found to be abundant in these patients, correlating positively with increased numbers of Vα24^+^ cells identified in the lung tissue [74].

The macrophage–iNKT interaction was also studied in other contexts of infection. In mice infected with *Bacillus anthracis*, α-GalCer administration delayed bacterial systemic dissemination and increased survival rate [43]. Interestingly, depletion of CD169^+^ macrophages from the subcapsular sinus correlated with impaired iNKT cell activation in the lymph nodes. This impairment was associated with enhanced *B. anthracis* dissemination out of the lymph nodes [43]. Moreover, to understand the interaction between macrophages and iNKT cells during enterovirus 71 (EV71) infection, experiments were performed in newborn mice, since this virus mainly affects infants [75]. In mice that were infected with EV71 at different days during the first 2 weeks of life, the survival rate was very poor when mice were infected before day 7, which was the day iNKT cells developed in this model. When mice were infected after iNKT cell development, the survival rate improved considerably. The later the day of infection, the better the survival rate [75]. Zhu et al. also correlated susceptibility to EV71 infection with low numbers of iNKT cells, and splenic and hepatic iNKT cells upregulated activation markers after EV71 activation. This activation was due to TLR3-dependent activation of macrophages rather than DCs and required endogenous CD1d ligands and IL-12 to reach their peak. In this mouse model, iNKT cells were also suggested to protect the central nervous system from EV71 dissemination and to be crucial antiviral effectors against EV71 infection before the adaptive immune system becomes fully functional in mice [75].

Lastly, the iNKT cell–macrophage crosstalk was also investigated in a human in vitro model of *Brucella suis* infection [76]. Human CD4^+^ iNKT cells were shown to impair the intramacrophagic growth of *Brucella suis* through several mechanisms. This effect was suggested to mainly depend on contact interactions between iNKT cells and macrophages, such as induction of the Fas pathway and release of lytic granules. Unlike IL-4 or TNF-α, IFN-γ also seems to have some relevance in this protective role. Evidence suggested CD4^+^ iNKT cells interact with macrophages via CD1d, eliminating bacteria or controlling their growth by killing their host, namely macrophages [76].

**Table 1:** Interaction between iNKT cells and macrophages in different contexts of infection.

Infection Model	Role	Readout	Ref.
CLP-induced sepsis	Protective	iNKT cells migrate from liver to peritoneum; iNKT cells increase macrophage phagocytic capacity.	[56]
CS-induced sepsis	Deleterious	PD-1-driven iNKT cells reduce macrophage activation.	[57]
CLP-induced sepsis	Deleterious	Lower numbers of hepatic iNKT cells; iNKT cells more activated; iNKT cells induce IL-6 secretion by hepatic macrophages.	[58]
*Candida albicans*	Deleterious	Activated iNKT cells correlated with lower survival rate; IL-10 production by iNKT cells.	[59]
*Candida albicans* post-sepsis	Deleterious	Impairment of macrophage phagocytic capacity by iNKT cells; Presence of iNKT cells associated with worst survival rate.	[60]
*Leishmania donovani*	Protective	Enhancement of hepatic iNKT cell activation in the presence of infected KCs; SIPRα-CD47 interaction enhances iNKT activation.	[61]
*Leishmania donovani*	Deleterious	Impairment of iNKT cell activation by infected splenic macrophages; GSPL-CD1d activates iNKT cells.	[62]
		GSPL-TLR4 activates macrophages.	[63]
*Borrelia burgdorferi*	Protective	KCs induce CXCR3-mediated iNKT cell clustering; iNKT cells reduced bacteria dissemination.	[64]
*Listeria monocytogenes*	Protective	iNKT cells and macrophages ameliorate listeriosis; α-GalCer-driven activation of iNKT cells enhances bactericidal functions of peritoneal macrophages.	[65]
*Mycobacterium tuberculosis*	Protective	Monocytes pulsed with α-GalCer lead iNKT cells to restrict Mtb growth.	[66]
*Mycobacterium tuberculosis*	Protective	iNKT cells can be activated by Mtb-infected macrophages via CD1d, IL-12 and IL-18; suppression of bacterial replication.	[67]
*Mycobacterium tuberculosis*	Protective	CD1d-dependent suppression of bacterial growth; iNKT cells secrete GM-CSF in the presence of Mtb-infected macrophages; GM-CSF is sufficient to control Mtb growth.	[68]
*Pseudomonas aeruginosa*	Protective	α-GalCer-driven activation of iNKT cells enhances phagocytosis by alveolar macrophages.	[69]
Influenza virus	Protective	iNKT cell activation induced by α-GalCer enhances early innate immune response; possible migration of hepatic iNKT cells to the lungs.	[71]
Influenza virus	Protective	iNKT cells were associated with better survival rate in infected mice.	[72]
Influenza virus	Protective	NKT activation results in an early wave of IL-4 secretion upon their relocation and priming of nearby resident macrophages; induction of IL-4 requires both CD1d and IL-18.	[73]
Sendai Virus	Deleterious	Inflammation is potentiated by IL-13 from iNKT-activated macrophages.	[74]
*Streptococcus pneumoniae*	Protective	Mrp1 deficiencies in macrophages: - Reduce CD1d clustering on the surface; - Induce lower activation of iNKT cells; - Increase mortality associated with the infection.	[70]
*Bacillus anthracis*	Protective	α-GalCer administration delays bacterial systemic dissemination and increases survival rate; depletion of CD169^+^ macrophages reduces iNKT activation and increases bacterial dissemination.	[43]
Enterovirus 71	Protective	iNKT cells increase survival rate; TLR3-dependent activation of iNKT cells by macrophages; iNKT activation also requires CD1d and IL-12.	[75]
*Brucella suis*	Protective	CD4^+^ iNKT cells impair bacterial intramacrophagic growth.	[76]

KCs: Kupffer cells; CLP: cecal ligation and puncture, CS: cecal slurry.

### 2.3. The iNKT Cell–Macrophage Axis in Obesity

iNKT cell–macrophage crosstalk has been extensively studied in the adipose tissue, where it was shown to influence the overall inflammatory status and ultimately modulate glucose homeostasis and insulin resistance processes, as summarized in Table 2 and Figure 1B [10,77,78,79]. The role of iNKT cells in obesity is not consensual, as they can exhibit protective or pathogenic roles in metabolic regulation and obesity-triggered inflammation. These alternative effects are partially dependent on the differential capacity of iNKT cells to modulate macrophage polarization towards a pro- or anti-inflammatory phenotype. In murine models of obesity fed with a high-fat diet (HFD), macrophages upregulated expression of M2 anti-inflammatory genes upon iNKT cell activation with α-GalCer [77,78]. Considering that this effect was abrogated in CD1d^−/−^ mice lacking iNKT cells [77,78], it seems that iNKT cells are crucial to induction of macrophage polarization towards an M2-skewed profile to inhibit metaflammation. iNKT-induced M2 polarization occurred following acute (4 days) or prolonged (8 weeks) HFD challenges in an IL-4-dependent manner [77,78]. Accordingly, adipose-tissue-resident regulatory iNKT cells were shown to trigger M2 polarization of macrophages in the absence of HFD challenge via IL-10 [54]. In addition, the number of iNKT cells in the adipose tissue was reported to be decreased in patients with obesity [80] and to inversely correlate with the percentage of pro-inflammatory macrophages infiltrating the adipose tissue [79].

However, others have proposed a pro-inflammatory role of iNKT cells in obesity. Zhang et al. reported an M2-specific CD1d downregulation in HFD-fed mice during obesity progression, which impaired the ability of M2 macrophages to present α-GalCer to iNKT cells, leading to Th1-like responses [81]. In addition, after 16 weeks of HFD feeding, iNKT cells preferentially interacted with M1 pro-inflammatory macrophages [81]. In a similar tendency, Ohmura et al. highlighted that HFD induced high rates of adipose-tissue iNKT infiltration and glucose intolerance, with α-GalCer-driven activation of iNKT cells significantly increasing infiltration of pro-inflammatory macrophages in the adipose tissue [82]. A similar M1-inducing effect has also been reported for hepatic iNKT cells activated by dietary lipids in HFD-fed mice exhibiting obesity-induced hepatic steatosis [83].

To further dissect how iNKT cells are implied in obesity, distinct iNKT-deficient animal models have been used. Some researchers have reported that β_2_-microglobulin, CD1d or Jα18 KO mice (which all lack iNKT cells) exhibit improved glucose homeostasis, protection against HFD-induced insulin resistance and decreased macrophage recruitment to the adipose tissue and liver [82,83]. However, others have observed no differences in weight gain, metabolic activity, insulin and glucose tolerance between HFD-fed CD1d^−/−^ mice and WT littermate controls [84].

Thus, the roles of macrophage–iNKT cell crosstalk in adipose tissue during obesity onset remain controversial. Duration of the HFD challenge seems to influence this interplay, considering that short-term HFD triggered higher infiltration of iNKT cells in the adipose tissue [78], unlike what is observed following longer periods of HFD feeding [77,80,83]. Hence, different stages of obesity apparently trigger distinct macrophage–iNKT cell interactions in the adipose tissue. In later stages of obesity, iNKT cells are suggested to play a more pathogenic role following activation by M1 macrophages to further promote inflammation and insulin resistance [81].

**Table 2:** Interactions between iNKT cells and macrophages in murine models of diet-induced obesity.

Model	Role	Readout	Ref.
60% HFD (4 days or 8 or 24 weeks)	Protective	M2 polarization of adipose tissue macrophages via IL-4 by activated iNKT cells.	[77,78]
60% HFD (6 or 12 weeks)	Protective	Inverse correlation between numbers of adipose tissue iNKT cells and pro-inflammatory macrophages.	[79]
HFD (1 or 3 days and 1, 4 or 12 weeks)	Deleterious	iNKT-mediated recruitment of pro-inflammatory macrophages into adipose tissue.	[83]
60% HFD (6, 8, 10 or 16 weeks)	Deleterious	iNKT activation by M1 macrophages exacerbated metaflammation and activation by M2-macrophage-ameliorated disease; M2-specific CD1d downregulation during obesity progression.	[81]

HFD: high-fat diet.

### 2.4. The iNKT Cell–Macrophage Axis in Sterile Inflammation

Critical interactions between iNKT cells and macrophages have also been pinpointed during sterile inflammation, mostly in the liver, peritoneum and intestine (Figure 1C). In a mouse model of local sterile liver injury, specialized KCs were shown to activate hepatic iNKT cells to promoting wound healing and tissue repair [85]. In this model, CD1d engagement, as well as IL-12 and IL-18, was required for iNKT cells to be arrested and activated near the injured site. IL-4 produced by activated iNKT cells promoted post-lesion hepatocyte proliferation, collagen deposition and monocyte reprogramming into a reparative CCR2^lo^CX3CR1^hi^ profile, thus culminating in resolution of the injury [85].

The interplay between KCs and hepatic iNKT cells has been further explored in mice fed with methionine/choline-deficient diets as a model of hepatosteatosis, a metabolic disease characterized by abnormal lipid accumulation in the liver [86,87]. Kremer et al. observed reduced hepatic iNKT cell numbers and IL-4 production accompanied by KC activation in mice fed with a 10-week choline-deficient diet (CDD) [86]. Notably, KC depletion by clodronate treatment restored hepatic iNKT cell numbers, suggesting that KCs were contributing to iNKT cell loss in the steatotic liver [86]. However, others have reported that feeding mice with a methionine and choline-deficient diet (MCD) for 3–5 days triggers iNKT cell recruitment and clustering in the mouse liver [87]. Following MCD challenge, CD1d-expressing KCs were shown to co-localize and interact with iNKT cells in cell clusters, contributing to lipid clearance by iNKT cells [87]. The contrasting differences in iNKT cell abundance in hepatosteatosis murine models observed in both studies may arise from the fact that different experimental approaches were used to induce murine hepatosteatosis and detect hepatic iNKT cells. Furthermore, considering that both studies differ in the duration of the dietary challenge, it is also possible that the stage of hepatosteatosis may influence the overall outcome of the iNKT cell–macrophage interactions in the steatotic liver.

Peritoneal iNKT cells and macrophages were also reported to interact in the context of acute sterile peritonitis induced by sodium periodate [88]. Peritoneal macrophages efferocytosing apoptotic neutrophils produced IL-4 and were capable of activating iNKT cells via CD1d to produce IL-4 and IL-13, thereby suppressing inflammation. CD1d and Jα18 KO mice exhibited abrogated cytokine production and exacerbated inflammation. Importantly, peritoneal inflammation diminished upon adoptive transfer of WT iNKT cells into Jα18^−/−^ but not CD1d^−/−^ mice, thus indicating that both the presence of iNKT cells and CD1d expression are required for this effect [88].

Growing evidence has highlighted that iNKT cells are central players in intestinal inflammation and promote the development of inflammatory bowel disease (IBD) [89]. It has been increasingly suggested that macrophages may act as modulators of these pathogenic roles of iNKT cells in IBD. Diphtheria-induced early-life depletion of embryonic macrophages diminished later-life iNKT cell infiltration in the barrier organs (skin and colon) and prevented oxazolone-induced colitis [50]. This is in accordance with earlier reports describing that colitis pathogenesis in this animal model is largely dependent on iNKT cells, essentially through IL-13 [90,91]. Likewise, although their role remains unclear, iNKT cells are also suggested to be implicated in airway hyper-reactivity (AHR) and asthma [92]. In mouse models of glycolipid-induced AHR, iNKT cells were shown to induce IL-33 production by alveolar F4/80^+^CD11c^−^ macrophages in a CD1d-restricted manner, which in turn activated and triggered IL-13 production by iNKT cells [93]. This IL-33- and IL-13-dependent loop of reciprocal activation between both cells lies at the base of airway inflammation within this animal model [93].

Some studies also describe that iNKT cells exacerbate atherosclerosis [94,95], with peritoneal macrophages pulsed with oxidized low-density lipoproteins prompting in vitro IFN-γ production by iNKT cells [94]. However, Smith et al. pinpointed an atheroprotective role of iNKT cells during early stages of atherosclerosis development in systemic lupus erythematosus (SLE) patients [96]. iNKT cells from SLE patients with asymptomatic atherosclerotic plaque exhibited an anti-inflammatory profile, and differentiation of healthy iNKT cells in the presence of serum from these patients triggered in vitro M2 polarization of THP-1 macrophages. In contrast, iNKT cells from SLE patients who had already suffered cardiovascular events exhibited low CD69 expression and failed to secrete IL-4 and IFN-γ ex vivo upon α-GalCer stimulation, which was associated with reduced frequencies of M2-like monocytes [96]. Hence, even though they promoted tolerance in early disease, iNKT cells became unresponsive in clinically advanced disease stages. Nevertheless, further research is required to better untangle the cumulative effects of iNKT cells and macrophages in atherogenesis.

### 2.5. The iNKT Cell–Macrophage Axis in Cancer

Macrophage–iNKT crosstalk in cancer has been scrutinized in recent decades. This crosstalk starts with cell migration and recruitment and ends with cell–cell interaction (Figure 1D). The mechanisms of chemoattraction bring iNKT cells and tumor-associated macrophages (TAMs) together in the tumor microenvironment, postulating the primary condition for cell–cell engagement, which is cell–cell co-localization. These mechanisms have been deeply understood in different mouse models. Monocytes were shown to be the only CD1d^+^ leukocytes co-localizing with iNKT cells, suggesting an interaction between these two subsets and their progeny in the tumor microenvironment [97]. Human iNKT cells were shown to migrate to neuroblastoma cells in response to CCL2 and to infiltrate tumors that highly express this chemokine [98]. Besides iNKT cells, CCL2 was also able to direct TAM precursors to the tumor site [99,100]. This evidence suggests a placement of both iNKT cells and TAM precursors in the tumor microenvironment, allowing their engagement, as reported by Song et al., in the context of primary neuroblastoma [101]. Although both cell subsets were shown to migrate to the tumor site, it is important to note that in xenograft mouse models for neuroblastoma, the frequency of tumor-infiltrated iNKT cells was drastically higher—up to 8 times—in the presence of TAMs, which were killed via CD1d [101]. These data reconcile with studies by Lui et al. that unveiled TAM-secreted CCL20 as another mediator for iNKT cell recruitment to the tumor microenvironment under hypoxic conditions [102] after CCL20 had been pointed out as a selective chemoattractant for DCs, effector-memory T cells [103] and NKT cells [104]. Interestingly, in this xenograft model, hypoxia inhibited the immunosurveillance of iNKT cells at the tumor site, which could be retrieved by IL-15 [102]. The combination iNKT/IL-15 presented potent and long-lasting antitumor activity in this model of metastatic neuroblastoma [102].

Given the preponderance of iNKT cells and macrophages at the tumor site [12], the details of the crosstalk cells might be of immense potential. Not only the direct effect on the tumor cells but also the capacity to reprogram and be reprogrammed comprise a set of crucial information to better understand the cell dynamics. In this line of thought, iNKT–macrophage interactions were studied in a prostate cancer mouse model [20]. It was verified that there were more infiltrating iNKT cells at the tumor site in comparison with normal tissue and that their absence led to an increase in the population of M2-like macrophages, which could be diminished upon iNKT cell transfer, delaying tumor progression. TAM populations were regulated by iNKT cells not only by protecting the M1 and killing the M2 but also by modulating the macrophage polarization preferentially into an M1 phenotype [20]. The associated mechanisms were partially unveiled as CD1d and Fas-FasL interactions required for iNKT cell-mediated M2-like macrophage death, while CD40 expression was sufficient for protection of M1-like macrophages from iNKT toxicity [20]. In a transgenic mouse model of melanoma, low numbers of intratumoral iNKT cells were found, and their antitumor function was impaired [105]. Activation of these cells was restored after α-GalCer treatment, significantly increasing the number of M1-macrophages in both spleen and tumor [105]. Besides their natural antitumor capacity, iNKT cells were also capable of inhibiting M2-like macrophages in a pancreas cancer model in a microsomal prostaglandin E synthase-1 (mPGES-1) and 5-lipoxygenase (5-LOX)-dependent manner. Interestingly, the pharmacological inhibition of these molecules in M2 macrophages decreased pancreatic lesions and enhanced the infiltration of active CD8^+^ cells [106]. Altogether, iNKT cells seem to contribute directly and/or indirectly to delayed tumor progression [20,105,106]. In contrast, results obtained in a colon adenocarcinoma transgenic mouse model demonstrated that iNKT cells promoted M2-like polarization of TAMs concomitantly with increased expression of FoxP3 protein and enhanced frequency of Tregs, assisting tumor progression and intestinal adenomatous polyp formation [107]. However, upon α-GalCer treatment, the frequency of M2-like splenic macrophages was reduced, whereas M1-like macrophage frequency increased [108]. Although colorectal cancer is frequently a distinct paradigm regarding macrophage and Treg expression, the mechanisms underlying this effect still require further elucidation.

The relevance of the macrophage–iNKT cell interplay has also been addressed in chronic lymphocytic leukemia (CLL) [109]. The addition of iNKT cells to a co-culture of CD14^+^ monocytes and CLL cells strongly impaired in vitro differentiation of monocytes into adherent nurse-like cells (NLC), which are CLL-specific TAMs. This effect required the engagement of CD1d. Although NLC cells were shown to sustain the survival of CLL cells, in the presence of iNKT cells, there were lower viability rates of both NLC and CLL cells, indicating that iNKT cells could affect CLL viability by indirectly restraining NLC via CD1d [109].

Overall, similar outcomes of iNKT–macrophage interactions are found in the context of cancer. Macrophages play a role in chemoattraction, driving iNKT cells to the tumor site. Most reports highlight an adaptive and innate antitumor capacity from iNKT cells that is supported and boosted by either α-GalCer or cytokine treatment, namely IL-12 and IL-15. When activated, iNKT cells were demonstrated, in several mouse models, to reduce infiltration of M2 macrophages and increase M1-like macrophage frequencies, leading to a better prognosis.

### 2.6. The iNKT Cell–Macrophage Axis in Autoimmunity

The macrophage–iNKT cell interplay has also been implicated in diverse autoimmune diseases, namely multiple sclerosis, type I diabetes and rheumatoid arthritis. In experimental autoimmune encephalomyelitis (EAE), a mouse model of multiple sclerosis (MS), α-GalCer-mediated iNKT cell activation resulted in decreased frequencies of Ly6C^hi^ inflammatory monocytes and polarization of CNS macrophages towards an M2-skewed profile [110]. These effects were dependent on both CD1d and IL-4. Strikingly, adoptive transfer of α-GalCer-conditioned M2-enriched monocytes into Jα18^−/−^ EAE mice successfully rescued neurological impairment and improved survival [110].

iNKT cells and macrophages are suggested to mediate opposing effects during type I diabetes (T1D) pathogenesis. Whereas several reports point to protective roles of iNKT cells in T1D, macrophages are strongly associated with pancreatic β cell destruction and T1D development [111], although few studies have focused on the direct communication between both cells in the context of T1D. Ghazarian et al. reported that infection with pancreatropic coxsackievirus B4 accelerated diabetes development and promoted infiltration of inflammatory macrophages into the pancreatic islets of non-obese diabetic (NOD) and proinsulin-2-deficient mice, both models of T1D [112]. Importantly, α-GalCer-driven activation of iNKT cells by the time of viral infection prompted macrophage expression of suppressive molecules, including indoleamine (IDO) 1 and 2 and arginase-1, via IFN-γ and IL-13. By selectively inhibiting IDO, the authors further showed that this iNKT-induced immunosuppressive pancreatic environment was required to prevent advanced insulitis and disease onset [112]. Hence, iNKT cells once again critically modulated macrophage phenotypes to mitigate inflammation.

Bimodal roles of iNKT cells have been described in rheumatoid arthritis (RA). Although the majority of studies point to iNKT cells contributing to joint inflammation and arthritis pathogenesis, others have described a suppressive effect of these cells in RA [113]. By using a murine model of collagen-induced arthritis (CIA), Miellot-Gafsou et al. observed that hepatic iNKT cells underwent activation during the early phase of the disease, with CD1d blocking in early but not in late disease stages decreasing CD40 expression on macrophages and DCs and ameliorating disease manifestations [114]. Contrarily, a recent report has underpinned that iNKT cells contribute to key immunosuppressive roles of peritoneal macrophages in CIA mice to promote immunological tolerance [115]. Although peritoneal macrophages from CD1d^+/−^ and CD1d^−^ mice did not register major differences in expression of distinct M1 and M2 cell surface markers, their cytokine profiles varied. Upon LPS stimulation, macrophages from CD1d^+/−^ mice exhibited a more anti-inflammatory profile, characterized by increased secretion of IL-10 and decreased secretion of TNF-α and IL-6. This further reflected in disease amelioration following adoptive transfer of CD1d^+/−^ peritoneal macrophages into CD1d KO mice with CIA [115].

Despite the reported dichotomous role of iNKT cells in autoimmunity, altogether, these results reveal that iNKT-mediated modulation of inflammation in autoimmune diseases is at least partially dependent on their capacity to regulate macrophage inflammatory profiles, as represented in Figure 1E.

## 3. Conclusions

Increasing evidence underscores the relevance of the iNKT cell–macrophage axis in several pathophysiological contexts. This information enlightens not only the details of iNKT cell activation upon macrophage engagement but also the modulation of macrophage functions and phenotypes after interaction with iNKT cells.

The rules and effects of this bimodal interplay depend on several parameters, such as cell types and tissue of origin, disease and developmental stage of the host immune system, thus featuring the complexity and importance of taking into consideration the context in which the cells interact. In this way, our current knowledge regarding iNKT–macrophage crosstalk can contribute to the establishment of better paradigms according to the microenvironment in which they occur. Since both iNKT cells and macrophages are highly heterogeneous, there is a need for comprehensive phenotypical analyses associated with the respective function set of the cell to optimally exploit the immunomodulatory properties of each subset.

This review covers the up-to-date state of the art on this topic. Altogether, the striking pieces of information herein discussed suggest that these cells stand as promising targets for the development of novel iNKT cell and/or macrophage-based immunotherapeutic strategies to prevent disease onset and progression.
